# Factors Associated with Spontaneous Clearance of Hepatitis C Virus in Chinese Population

**DOI:** 10.1155/2014/527030

**Published:** 2014-07-16

**Authors:** Fei Kong, Yu Pan, Xiumei Chi, Xiaomei Wang, Linjiao Chen, Juan Lv, Haibo Sun, Ruihong Wu, Jinglan Jin, Ge Yu, Zhenhua Ma, Yang Wang, Xinxing Huang, Hua Li, Yang Bai, Jing Jia, Gerald Y. Minuk, Jin Zhong, Bing Sun, Jing Jiang, Junqi Niu

**Affiliations:** ^1^Department of Clinical Epidemiology, First Hospital of Jilin University, 71 Xin Min Street, Changchun, Jilin Province 130021, China; ^2^Department of Hepatology, First Hospital of Jilin University, 71 Xin Min Street, Changchun, Jilin Province 130021, China; ^3^Key Laboratory of Zoonoses Research, Ministry of Education, 519 Dong Minzhu Street, Changchun, Jilin Province 130021, China; ^4^Department of Infection, Affiliated Hospital of Beihua University, 71 Liberation Road, Jilin City, Jilin Province 132011, China; ^5^Ultrasound Department of Hepatology, First Hospital of Jilin University, 71 Xin Min Street, Changchun, Jilin Province 130021, China; ^6^Section of Hepatology, University of Manitoba, Winnipeg, MB, Canada R3T 2N2; ^7^Institute Pasteur of Shanghai, Shanghai Institutes for Biological Sciences, Chinese Academy of Sciences, 320 Yue Yang Street, Shanghai 200031, China

## Abstract

Hepatitis C virus (HCV) infections spontaneously clear in approximately 15–45% of infected individuals. Factors which influence spontaneous HCV clearance remain to be identified. The purpose of the present study was to identify variables associated with spontaneous HCV clearance in a referred population of Chinese patients. The prevalence of host, viral, and environmental factors known to influence the outcome of HCV infections was compared in 92 HCV spontaneous clearance subjects and 318 HCV persistent infection subjects. Univariate and multivariate analyses were performed to identify those factors associated with spontaneous HCV clearance. In univariate analysis, female gender, a history of icteric hepatitis, serologic evidence of concurrent HBV infection, and rs12979860 CC genotype were positively associated with spontaneous HCV clearance, while alcohol consumption was negatively associated with clearance. In multivariate analysis, female gender, a history of icteric hepatitis, concurrent HBV infection, and rs12979860 CC genotype remained independent variables associated with spontaneous HCV clearance. Spontaneous HCV clearance is more likely to occur in females, subjects with a history of icteric hepatitis, HBV coinfections, and those with the rs12979860 CC genotype.

## 1. Introduction

Hepatitis C virus (HCV) infection is an important global health problem with approximately 130–210 million individuals or 2.2–3% of the world's population being infected [1–3]. In China, the prevalence of HCV is approximately 3.2% [4].

There are two distinct outcomes of acute HCV infection: spontaneous viral clearance (occurring in approximately 15–45% of subjects) and progression to chronic infection (55–85% of subjects) [5–9]. Patients who spontaneously clear HCV can be identified by testing positive for antibody to HCV (anti-HCV) in the absence of detectable HCV-RNA [10]. In those who develop chronic infections (anti-HCV positive/HCV-RNA positive) progression to fibrosis, cirrhosis, end stage liver disease, and hepatocellular carcinoma can occur [9, 11–14].

Clinical outcomes of HCV infection are influenced by the interplay between host, viral, and environmental factors. Recently, female gender, a young age at infection, aboriginal ethnicity, and a history of icteric hepatitis were reported to be associated with higher spontaneous HCV clearance rates [15, 16], while African-American ethnicity and excess alcohol consumption were associated with low viral clearance rates [17, 18]. Coinfection with hepatitis B (HBV) and hepatitis A (HAV) viruses has also been associated with significantly higher spontaneous HCV clearance rates, while coinfection with the human immunodeficiency virus (HIV) is associated with low clearance rates [9, 17, 19–21]. In addition, genetic variations in* IL28B* appear to play an important role in spontaneous HCV clearance [12, 19, 20].

Because acute HCV infection is commonly a mild or asymptomatic illness [14, 22], the precise date of infection is often unknown. As a result, the natural history of HCV and determinants of viral clearance have not been fully elucidated. In the only study published to date amongst Chinese patients, spontaneous HCV clearance was documented in 20% of paid plasma donors and more commonly in female donors [23].

The purpose of the present study was to enhance our understanding of the natural history of HCV infection and identify those variables associated with spontaneous HCV clearance.

## 2. Subjects and Methods

### 2.1. Study Population

The study population was derived from 2,021 Chinese patients who participated in a retrospective investigation of HCV infection which was performed by the First Hospital of Jilin University in Fuyu County, Jilin province, from November 2009 to September 2011. Five hundred forty-seven of these subjects tested positive for anti-HCV and negative for HIV. The majority (64%) of HCV infections were ascribed to elicit injection drug use of caffeine and sodium benzoate (CNB) using shared syringes in the 1980s. One hundred thirty-seven individuals had received antiviral treatment for HCV infection and were therefore excluded from analysis. The remaining 410 subjects (285 male and 125 female) constituted the study population. Of these, 92 individuals were anti-HCV positive and negative for HCV-RNA on at least two occasions, a minimum of six months apart (spontaneous clearance). The remaining 318 individuals were both anti-HCV and HCV-RNA positive (persistent infection) with the latter being repeatedly positive over at least a 6-month period of testing.

This study was approved by the Ethical Committee at the First Hospital of Jilin University (Changchun, China) and informed consent was obtained from each subject.

### 2.2. Data Collection

On referral, each subject had been requested to complete a questionnaire that included information on demographics, risk factors for HCV infection, clinical features, behavioral activity, and family history. Demographic information included age, gender, and ethnicity. Clinical information included the presence or absence of diabetes, thyroid disease, autoimmune disorders and previous surgeries, blood transfusions, blood donations, and antiviral treatments. Behavioral activity included questions regarding injection drug use (nature of the drugs injected, frequency, and date of first/last injection), alcohol abuse, and smoking. The age at infection was considered to be the subject's age when elicit injection drug use first occurred. The questionnaire also contained information regarding family histories of liver disease.

### 2.3. Virological Testing

Serological markers for anti-HCV, antibody to hepatitis B surface antigen (anti-HBs), and HIV were determined by an enzyme-linked immunosorbent assay, using Abbott ARCHITECT i2000SR (Abbott Laboratories, Abbott Park, IL, USA). Anti-HCV positive results were confirmed by recombinant immunoblot assay (CHIRON RIBA HCV 3.0 SIA, Ortho Clinical Diagnostics, Johnson & Johnson, USA) in individuals who subsequently tested negative for HCV-RNA. HCV-RNA testing was performed by the Roche Taqman HCV test (Roche Diagnostics, Grenzach, Germany). Virological testing was repeated six months after the initial test.

### 2.4. IL28B Genotyping

Genomic DNA was isolated from peripheral blood cells using a genomic DNA purification kit (Promega Co., USA). Genotyping of the rs12979860 was performed (in 90 subjects with evidence of spontaneous clearance and 312 with persistent infections) by a pyrosequencing method, according to the manufacturer's protocol (PyroMark ID Pyrosequencing machine, QIAGEN). PCR primers for rs12979860 were forward: 5′-ATTCCTGGACGTGGATGGGTAC-3′, reverse: 5′-biotin-AGCGCGGAGTGCAATTCA-3′.

### 2.5. Statistical Analysis

Continuous variables were presented as median (interquartile ranges) or mean (SD) and compared by the Mann-Whitney test or two-sample *t*-test. Categorical variables were expressed as numbers and frequencies (%) and compared by the Pearson chi-square test or Fisher exact test. The unconditional logistic regression model was used to evaluate factors potentially associated with spontaneous HCV clearance. Hardy-Weinberg equilibrium was assessed by a goodness-of-fit *χ* ^2^ test. Variables with *P* values below or equal to 0.10 in univariate analysis were included in the multivariate model. Odds ratio (OR) and 95% confidence intervals (95% CI) were used to determine the strength of statistical associations. A two-sided *P* value below or equal to 0.05 was considered significant. All statistical analyses were performed using SPSS software package 18.0 (SPSS Inc. USA).

## 3. Results

### 3.1. Features of the Study Populations

Features of the two study populations and differences in the variables potentially associated with HCV outcomes are shown in Table 1. The mean ages of patients with spontaneous HCV clearance versus those with persistent infections were similar (50.6 versus 50.9 years, *P* = 0.824). Patients with spontaneous HCV clearance were more often females (44.6% versus 26.4%, *P* = 0.001) and less often provided a history of alcohol use (43.5% versus 59.4%, *P* = 0.007). There was no statistically significant difference in current alcohol use between the two cohorts (*P* = 0.425). Estimated ages at infection were also similar in the two groups (spontaneous HCV clearance cohort: 25.0 years versus persistent HCV cohort: 23.6 years, *P* = 0.318) as were the percent of individuals under the age of 20 years at the estimated time of infection (19.0% versus 31.6%, resp., *P* = 0.103). Estimated durations of infection were almost identical in the two groups (27.8 versus 27.6 years, resp.). The spontaneous HCV clearance group was more likely to have a history of acute icteric hepatitis than those with chronic infections (18.5% versus 7.2%, *P* = 0.001) and the prevalence of HBV coinfection was higher in those with spontaneous HCV clearance (20.7% versus 3.8%, resp., *P* < 0.001). Finally, the frequencies of gene polymorphisms of* IL28B* were investigated in 312 subjects with persistent infection and 90 individuals who spontaneously cleared HCV. The genotype distributions were in Hard-Weinberg equilibrium (*P* = 0.202). Subjects with the CC genotype of rs12979860 were more likely to spontaneously clear HCV than those with the CT genotype (*P* = 0.018). HCV-RNA genotyping was performed on those with chronic infections. Here, genotype 1b was detected in 176/318 (55.4%), genotype 2a in 117/318 (36.8%), and genotypes 1b and 2a coinfection in 6/318 (1.9%). There were 19 chronically infected subjects in whom HCV-RNA genotyping was not possible due to low HCV-RNA levels.

### 3.2. Univariate and Multivariate Analysis of Variables Associated with Spontaneous HCV Clearance

The results of univariate and multivariate analysis are provided in Table 2. According to univariate analysis, female gender (OR = 2.24, 95% CI: 1.39–3.62), a history of acute icteric hepatitis (OR = 2.91, 95% CI: 1.48–5.72), serologic evidence of HBV coinfection (OR = 5.78, 95% CI: 2.65–12.62), and the rs12979860 CC genotype (OR = 3.34, 95% CI: 1.17–9.60) were associated with an increased probability of spontaneous HCV clearance while a history of alcohol consumption was associated with a lower probability of spontaneous clearance (OR = 0.53, 95% CI: 0.33–0.84). After adjusting for potential confounders, female gender (OR = 2.18, 95% CI: 1.13–4.21), a history of acute icteric hepatitis (OR = 3.40, 95% CI: 1.68–6.88), rs12979860 CC genotype (OR = 3.29, 95% CI: 1.12–9.72), and the presence of HBV coinfection (OR = 5.51, 95% CI: 2.43–12.52) were the only four variables independently associated with spontaneous HCV clearance on multivariate analysis.

## 4. Discussion

The results of the present study confirm previous reports that female gender [4], a history of acute icteric hepatitis [20], serologic evidence of HBV coinfection [16, 21, 24, 25], and the rs12979860 CC genotype [12, 19, 26–28] are variables associated with spontaneous HCV clearance. However, they do not support previous reports that other variables such as age at infection [29] and alcohol use [17] serve as independent predictors of such an outcome.

Why spontaneous HCV clearance rates are higher in females than males remains to be determined. Based on better responses to interferon-*α* treatment in young females compared to older women or men, Hoyoshi et al. proposed that high levels of estrogen may contribute to HCV clearance [30]. In support of this possibility are data that the most potent physiological estrogen, 17 beta-estradiol, inhibits HCV replication in an Er*α*-dependent manner in vitro [31]. Clearly, further research in this important area is warranted.

The finding that subjects with a history of acute icteric hepatitis were 3.4-fold more likely to spontaneously clear HCV is in accordance with results of a study reported by Barrett et al. [20]. In their study, Irish women who had been infected with HCV following the administration of contaminated anti-D immunoglobulin were more likely to spontaneously clear HCV if they developed acute icteric hepatitis than those who remained anicteric. Similar results were observed in another Irish study by Sachithanandan and Fielding [32]. Unfortunately, the design of the present study did not allow determinations as to whether the episode of acute icteric hepatitis coincided with HCV clearance or represented an unrelated event. If the former, presumably the icterus reflected a more robust immune response to the virus, resulting in more extensive hepatic injury and the development of icterus or jaundice.

Coinfection with other hepatotropic viruses has also been shown to influence the natural history of HCV infections [9, 17, 19–21]. In the present study, we found that patients with serologic evidence of HBV coinfection were 5.51-fold more likely to have spontaneously cleared HCV than those with HCV infections alone. Similar results were described in Chinese blood donors and other patient populations [16, 21, 24, 25]. The mechanism responsible for this finding may reflect the increased expression of interferon-*γ* and TNF-*α* by host inflammatory cells in response to a superimposed HBV infection [33]. In support of this possibility are data describing inhibition of HCV replication in subjects with acute hepatitis A infections [21]. On the contrary, HIV infection is likely to interfere with spontaneous HCV clearance as a result of its immunosuppressive properties [19].

Our data demonstrated that the rs12979860 polymorphism near* IL28B* locus was also associated with enhanced spontaneous HCV clearance. This finding is consistent with recent reports describing* IL28B* gene variance including rs12979860 and rs8099917 being associated with high rates of spontaneous HCV clearances in Caucasian, African-American, and Chinese population [12, 19, 26–28]. In a related study, subjects with protective rs12979860 CC genotype tended to have higher serum Type III interferon levels (including IL28A, IL28B, and IL29), which possess potent antiviral properties [12, 26, 34].

Some studies have suggested that HCV clearance rates tend to be higher in younger individuals [29]. Related to this finding is a study by Zhang et al. who described enhanced HCV clearance amongst hemophiliac patients under the age of 2 years when compared to subjects who are 16 years of age or older [15]. While we did not find a significant association between estimated age at infection and HCV clearance, our study largely involved adults and, therefore, the number of young individuals was limited. Moreover, due to the retrospective study design, the age of infection could not be precisely ascertained. Nonetheless, our results are in keeping with those described by Bucsh et al. in adult blood donors [35].

Presumably, as a result of its immunosuppressive properties, alcohol consumption is thought to interfere with HCV clearance [17]. Although our data do not support that possibility, the amount of alcohol consumed is likely to be relevant as is the timing of consumption relative to viral exposure, two factors that our questionnaire could not capture. It should be noted, however, that other groups also failed to identify an association between alcohol consumption and HCV clearance [23].

The present study has certain additional limitations that warrant emphasis. First, due to the retrospective study design, it was not possible to determine whether HCV infections antedated or postdated HBV infections. Second, anti-HBc and HBV-DNA testing was not performed and, therefore, evidence of HBV coinfections over the estimated 30 years of HCV infection would have been underestimated. Third, we did not detect the presence or absence of HCV-RNA in liver tissue, and uncertainty exists as to whether the HCV-RNA clearance subjects truly resolved their HCV infection or actually have current infection undetectable in blood but detectable in liver. Finally, the variables identified in this trainer set of patients were not tested prospectively in a validation set.

## 5. Conclusions

In the present study, female gender, a history of icteric hepatitis, HBV coinfection, and the rs12979860 CC genotype are variables associated with spontaneous HCV clearance in this Chinese population. These findings illustrate the importance of host and viral determinants in the natural history of HCV infections. A better understanding of the natural history of HCV infections should lead to improving therapeutic strategies, economic models, and health-care policy decisions.

## Figures and Tables

**Table 1:** Features of HCV clearance and persistence subjects.

Characteristic	HCV clearance (*n* = 92)	HCV persistence (*n* = 318)	*P* value
Age (ys, SD)	50.6 (9.1)	50.9 (7.7)	0.824
Female (%)	41 (44.6)	84 (26.4)	0.001
Smoking history (%)	58 (63.0)	209 (65.7)	0.635
Alcohol consumption			
Past alcohol use (%)	40 (43.5)	189 (59.4)	0.007
Current alcohol use (%)	33 (35.9)	100 (31.4)	0.425
Age at infection (ys, SD)	25.0 (7.0)	23.6 (8.3)	0.318
Age at infection (<20 ys) (%)	8 (19.0)	67 (31.6)	0.103
Duration of infection (ys) (SD)	27.8 (6.6)	27.6 (7.5)	0.896
Icteric hepatitis history (%)	17 (18.5)	23 (7.2)	0.001
HBV coinfection (%)	19 (20.7)	12 (3.8)	<0.001
rs12979860			
CC (%)	86 (95.6)	270 (86.5)	0.018
CT (%)	4 (4.4)	42 (13.5)	

**Table 2:** Univariate and multivariate analysis of factors associated with HCV spontaneous clearance.

Characteristic	Univariate analysis (*n* = 410)		Multivariate analysis (*n* = 410)	
	OR (95% CI)	*P* value	OR (95% CI)	*P* value
Icteric hepatitis history				
No (%)	Reference	0.002	Reference	0.001
Yes (%)	2.91 (1.48–5.72)		3.40 (1.68–6.88)	
HBV coinfection				
HBsAg negative (%)	Reference	<0.001	Reference	<0.001
HBsAg positive (%)	5.78 (2.65–12.62)		5.51 (2.43–12.52)	
Sex				
Male (%)	Reference	0.020	Reference	0.020
Female (%)	2.24 (1.39–3.62)		2.18 (1.13–4.21)	
Alcohol consumption				
No (%)	Reference	0.007	Reference	0.612
Yes (%)	0.53 (0.33–0.84)		0.85 (0.45–1.60)	
Rs12979860 (*n* = 403)				
CT (%)	Reference	0.025	Reference	0.031
CC (%)	3.34 (1.17–9.60)		3.29 (1.12–9.72)	
